# Development of droplet digital PCR for the detection of *Tilletia laevis*, which causes common bunt of wheat, based on the SCAR marker derived from ISSR and real-time PCR

**DOI:** 10.1038/s41598-020-72976-7

**Published:** 2020-09-30

**Authors:** Tongshuo Xu, Zhaoqun Yao, Jianjian Liu, Han Zhang, Ghulam Muhae Ud Din, Sifeng Zhao, Wanquan Chen, Taiguo Liu, Li Gao

**Affiliations:** 1 State Key Laboratory for Biology of Plant Disease and Insect Pests, Institute of Plant Protection, Beijing, 100193 China; 2 Key Laboratory at Universities of Xinjiang Uygur Autonomous Region for Oasis Agricultural Pest Management and Plant Protection Resource Utilization, Shihezi University, Xinjiang, 832003 China; 3 School of Agriculture, Yangtze University, Hubei, 434023 China

**Keywords:** Microbiology, Fungi, Pathogens

## Abstract

Common bunt of wheat caused by *Tilletia laevis* and/or *T. caries* (syn. *T. tritici*)*,* is a major disease in wheat-growing regions worldwide that could lead to 80% or even total loss of production. Even though *T. laevis* can be distinguished from *T. caries* on the bases of morphology of teliospores using microscopy technique. However, molecular methods could serve as an additional method to quantify the pathogen*.* To develop a rapid diagnostic and quantify method, we employed the ISSR molecular marker for *T. laevis* in this study. The primer ISSR857 generated a polymorphic pattern displaying a 1385 bp *T. laevis-*specific DNA fragment. A pair of specific primers (L57F/L57R) was designed to amplify a sequence-characterized amplified region (SCAR) (763 bp) for the PCR detection assay. The primers amplified the DNA fragment in the tested isolates of *T. laevis* but failed in the related species, including *T. caries*. The detection limit of the primer set (L57F/L57R) was 5 ng/µl of DNA extracted from *T. laevis* teliospores. A SYBR Green I real-time PCR method for detecting *T. laevis* with a 100 fg/µl detection limit and droplet digital PCR with a high sensitivity (30 fg/µl detection limit) were developed; this technique showed the most sensitive detection compared to the SCAR marker and SYBR Green I real-time PCR. Additionally, this is the first study related the detection of *T. laevis* with the droplet digital PCR method.

## Introduction

Common bunt of wheat is a major disease worldwide that is caused by *Tilletia laevis* and (or) *T. caries* (syn. *T. tritici*)^1^. The pathogens produce teliospores in kernels, which are usually called “bunt balls”. The teliospores have an undesirable flavor and taste in wheat and flour, and thus, the disease not only causes yield reduction but also reduces the quality of wheat grain; even in wheat flour, teliospores of the pathogen still exist^2^. To date, the main control method is seed treatment with pesticides, and there are no effective and environmentally friendly pesticides for seed treatment^3^. In addition, *T. controversa* and *T. indica* are quarantine organisms in many countries^4,5^, while *T. caries* and *T. laevis* are widely distributed globally.

To date, the major detection methods of the pathogens of *T. laevis* and *T. caries* have been based on teliospore morphology^6^, triacylglycerol features^7^, immunological methods^8^, polypeptide profiles^9^ and genetic properties^10^. All these methods were difficult to handle, which require special skill and equipment. Molecular diagnosis technology is a low labor-requiring, efficient tool for the identification of fungal species^11^. Several studies have tried to identify specific markers for *Tilletia* species based on ITS, IGS1, and RPB2, but their results are not satisfactory^12,13^. However, rep-PCR fingerprinting, RAPD primer-mediated asymmetric PCR (RM-PCR), and sequence-characterized amplified region (SCAR) markers based on amplified fragment length polymorphism (AFLP) and inter-simple sequence repeat (ISSR) have been employed to successfully distinguish *T. controversa* from its related species^14–18^. Hence, DNA marker technology may be a powerful tool to distinguish *T. laevis* from other related species, especially on quantification aspects.

Compared to common PCR, real-time PCR is better with a high degree of sensitivity, specificity, repeatability, and reliability^19–21^, and it does not need to run gels after the reaction, save and time and eliminate the possibility of contamination. Real-time PCR is very popular in high throughput detection and quantification^22^. While, real-time PCR also has some limits^23^, such as a standard curve based on known concentration of target is necessary for getting the output data into actual values, and low accuracy of quantification will influence Cq value^24^.

However, droplet digital PCR (ddPCR), which is a sensitive technology that can amplify a highly diluted single molecule in a droplet, has the potential to improve the abovementioned limitations of real-time PCR, and the target pathogen can be detected by a fluorescent labeling probe^25,26^. Additionally, ddPCR can measure the absolute quantity of the pathogen without external nucleic acid standards. Without the need for standards, based on Poisson’s distribution, positive and negative compartments are counted, and the absolute concentration of target copies in the initial sample can be determined^27^. Moreover, the final result is independent of variations in the PCR amplification efficiency, indicating that ddPCR may be more accurate, have higher repeatability, and be less prone to interlaboratory variations than real-time PCR^28^. The ddPCR method distributes the sample into thousands of independent nanoscale droplets, which removes the issues with inhibition, minimizes the deviation of reaction factors in the target samples, has the ability to accurately identify the target molecules in the presence of sufficient nontarget molecules and can calculate the accurate and original concentration of the target molecule^29^. The ddPCR method has been used for quantification, molecular identification, and evolutionary analysis; increases the amplification efficiencies and can detect the lowest concentration of the nucleic acid in the molecules^25,30^. Some reports have also mentioned that ddPCR can be more resilient to inhibitors than its non-digital counterpart^29,31,32^. Recently, ddPCR methods were successfully developed for *T. controversa*, a similar pathogen, with high sensitivity^33^. To date, there have been no studies using this technique for the detection of the teliospores of *T. laevis*.

Until now, Zhang et al. developed an AFLP-derived SCAR marker (286 bp) for *T. laevis*, but they only tested a limited number of similar strains and did not mention the detection limit of the SCAR marker^19^. Yao et al. developed an ISSR-derived SCAR marker (660 bp) for *T. laevis* with a detection limit of 0.4 ng/μl of DNA from *T. laevis*, and they also developed a SYBR Green I real-time PCR method based on the SCAR marker with a detection limit of 10 fg/μl of *T. laevis* DNA^20^. In this study, we developed a rapid and accurate method for SCAR marker detection in *T. laevis*, and based on the SCAR marker, we also reported that real-time PCR and droplet digital PCR with high sensitivity contribute to accurate detection. Additionally, this is the first study related to detecting the teliospore of *T. laevis* with the high-sensitivity ddPCR method.

## Results

### Specific ISSR marker screening and SCAR marker development

**Figure 1:**
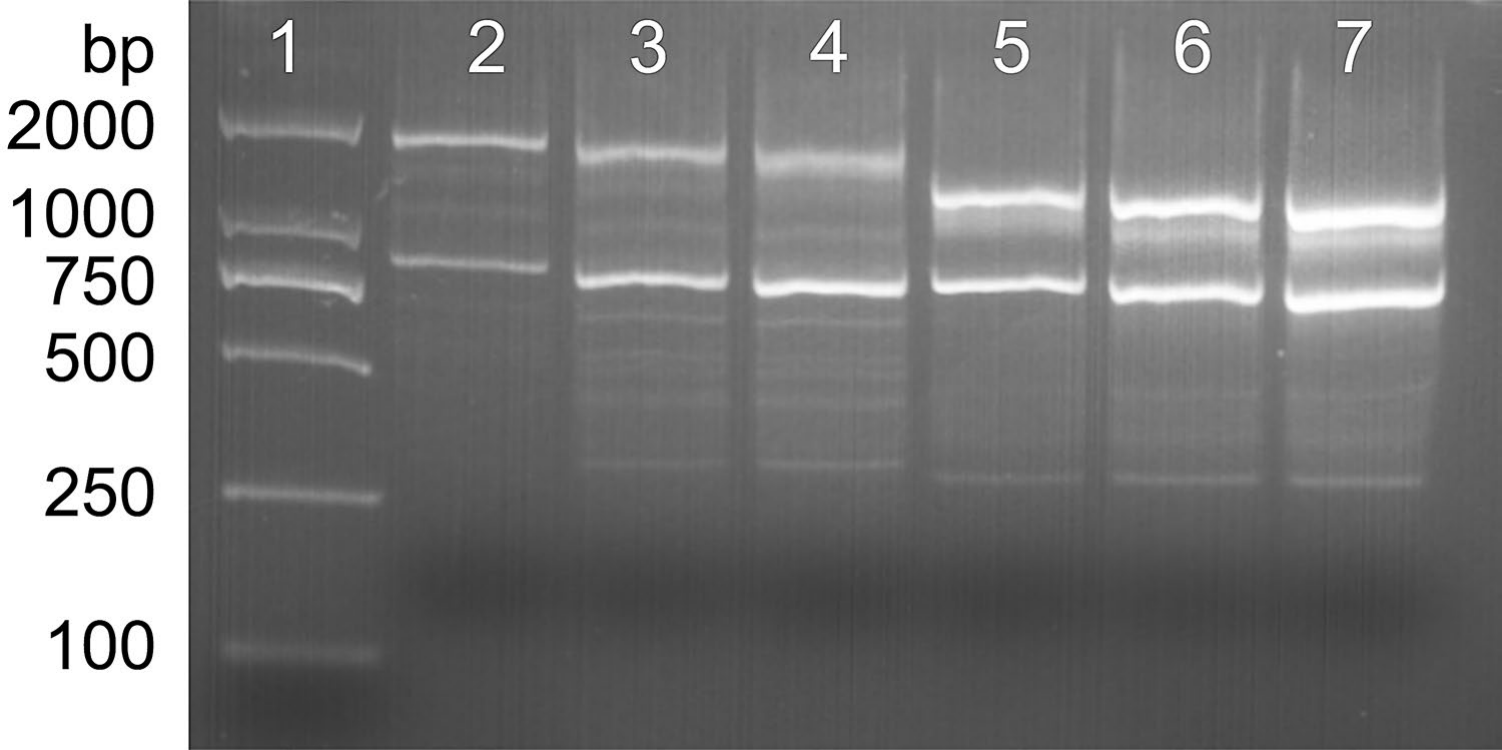
Specific fragment of *T. laevis* with an inter-simple sequence repeats (ISSR857) primer. Lane 1: DL2000 DNA ladder, Lanes 2–4: *T. laevis*, Lanes 5–7: *T. controversa*.

**Figure 2:**
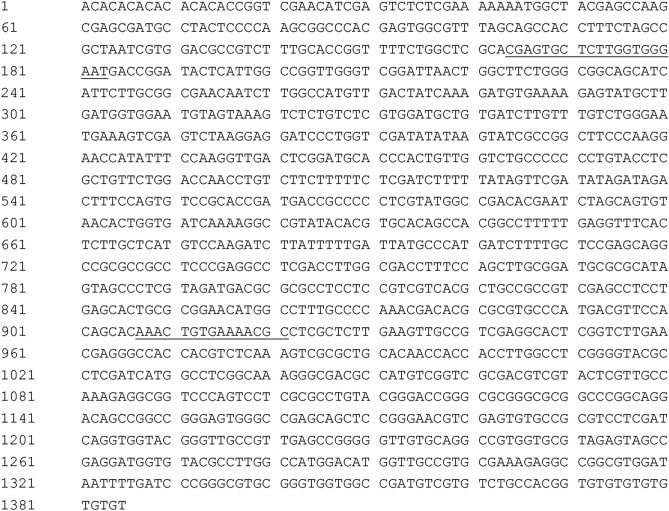
Sequence of a specific DNA fragment of *Tilletia laevis*. The sequence used for the amplification primers (L57F and L57R) is underlined.

From 100 ISSR primers, the primer ISSR857 (5´-ACACACACACACACA-3´) produced a polymorphic profile (1385 bp) only in *T. laevis* and no polymorphic profile in any of the other investigated pathogens (Fig. 1). Based on the specific DNA sequence of *T. laevis* (Fig. 2), the SCAR pair of primers named L57F (5′-CGAGTGCTCTTGGTGGGAAT-3′) and L57R (5′-GCGAGGCGTTTTCACAGTTT-3′) was designed by Primer Premier 5 for *T. laevis.* The primers amplified a 763 bp fragment from *T. laevis.*

### Specificity and sensitivity of the SCAR marker

**Figure 3:**
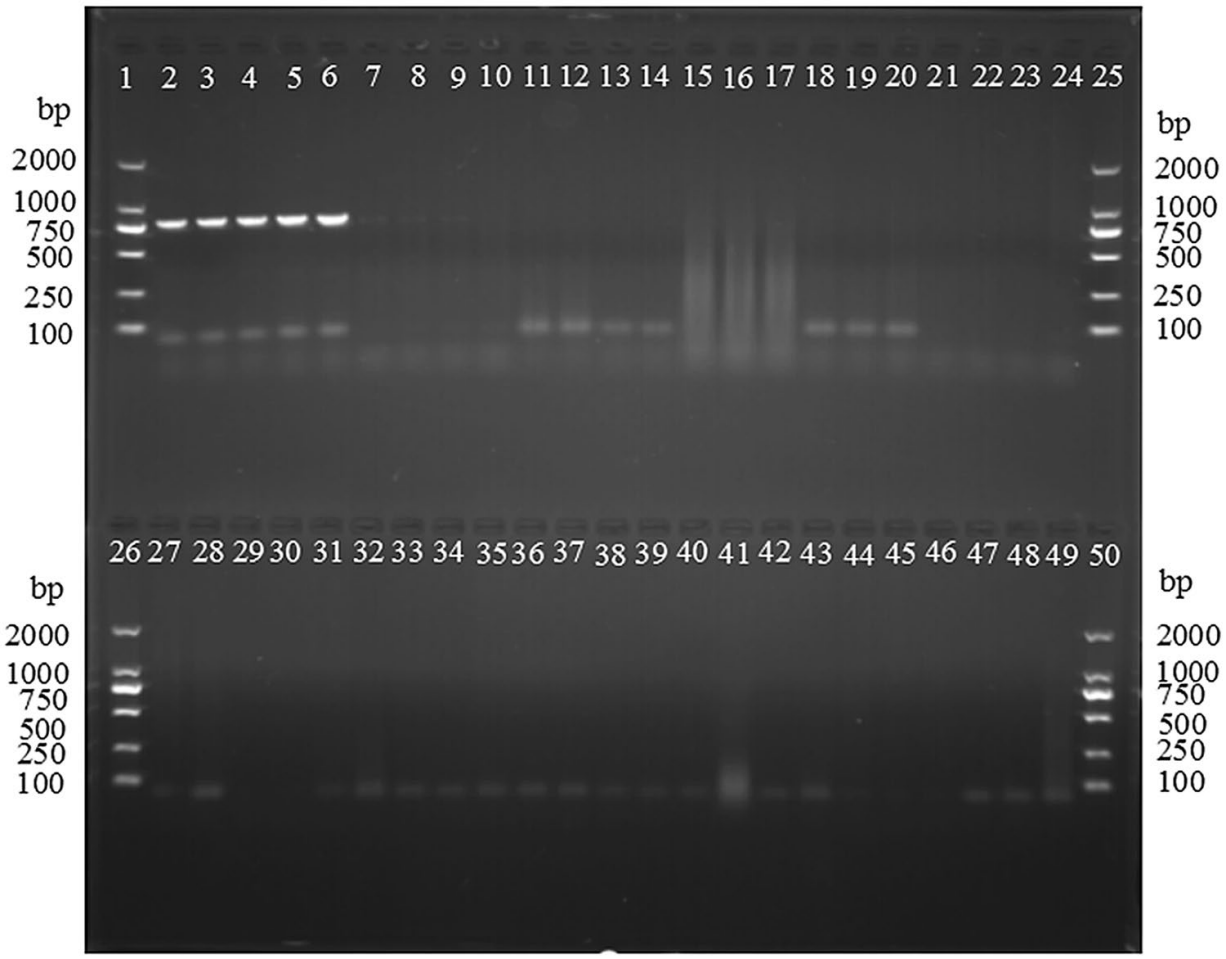
PCR with SCAR primers (L57F/L57R) to amplify genomic DNA from *Tilletia laevis* and other species. Lanes 2–6: *T. laevis,* Lanes 7–9: *T. controversa,* Lanes 10–12: *T. caries*, Lanes 13–16: *Ustilago tritici*, Lanes 17–19: *Puccinia striiformis.* f. sp. *tritici*, Lanes 20–22*: U. hordei*, Lanes 23–24, Lanes 27–28: *P. triticina*, Lanes 29–33: *U. maydis*; Lanes 34–36: *Rhizoctonia cerealis*, Lanes 37–38*: Fusarium graminearum*, Lanes 39–42: *Bipolaris sorokiniana,* Lanes 43–45: *P. graminis* f. sp. *tritici*, Lanes 46–48: *Blumeria graminis* f. sp. *tritici*; Lane 49: ddH_2_O, Lanes 1, 25, 26, 50: marker D2000.

**Figure 4:**
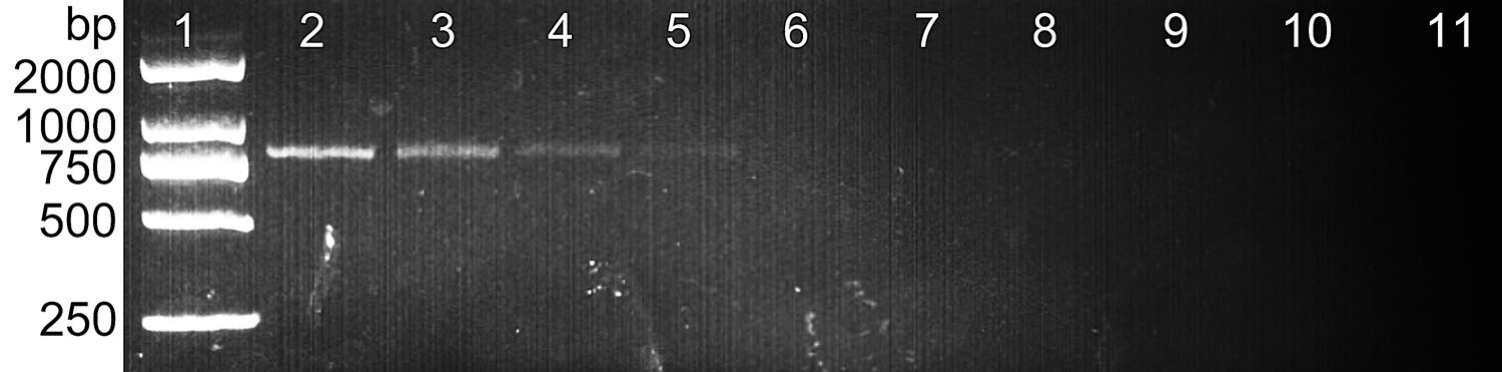
The sensitivity of the SCAR markers (L57F/L57R) with different amounts of DNA template in a 25 µl PCR mixture. Lane 1: DL500 DNA ladder, Lane 2: 50 ng/µl, Lane 3: 25 ng/µl, Lane 4: 10 ng/µl, Lane 5: 5 ng/µl, Lane 6: 1 ng/µl, Lane 7: 100 pg/µl, Lane 8: 10 pg/µl, Lane 9: 1 pg/µl, lane 10: 0.1 pg/µl, and lane 11: ddH_2_O.

In Fig. 3, the SCAR primer amplified a specific 763 bp fragment only from *T. laevis* and not the other tested pathogens (*T. laevis, T. controversa, T. caries, Ustilago tritici, U. hordei, U. maydis, Puccinia striiformis* f. sp. *tritici, P. graminis* f. sp*. tritici, P. triticina, Rhizoctonia cerealis, Fusarium graminearum*, *Blumeria graminis* f. sp. *tritici* and *Bipolaris sorokiniana*). The sensitivity of the SCAR marker was tested using a series of dilutions of the genomic DNA of *T. laevis*. The results showed that the sensitivity of the primers L57F/L57R was 5 ng of DNA in a 25 µl PCR mixture (Fig. 4).

### Real-time PCR

**Figure 5:**
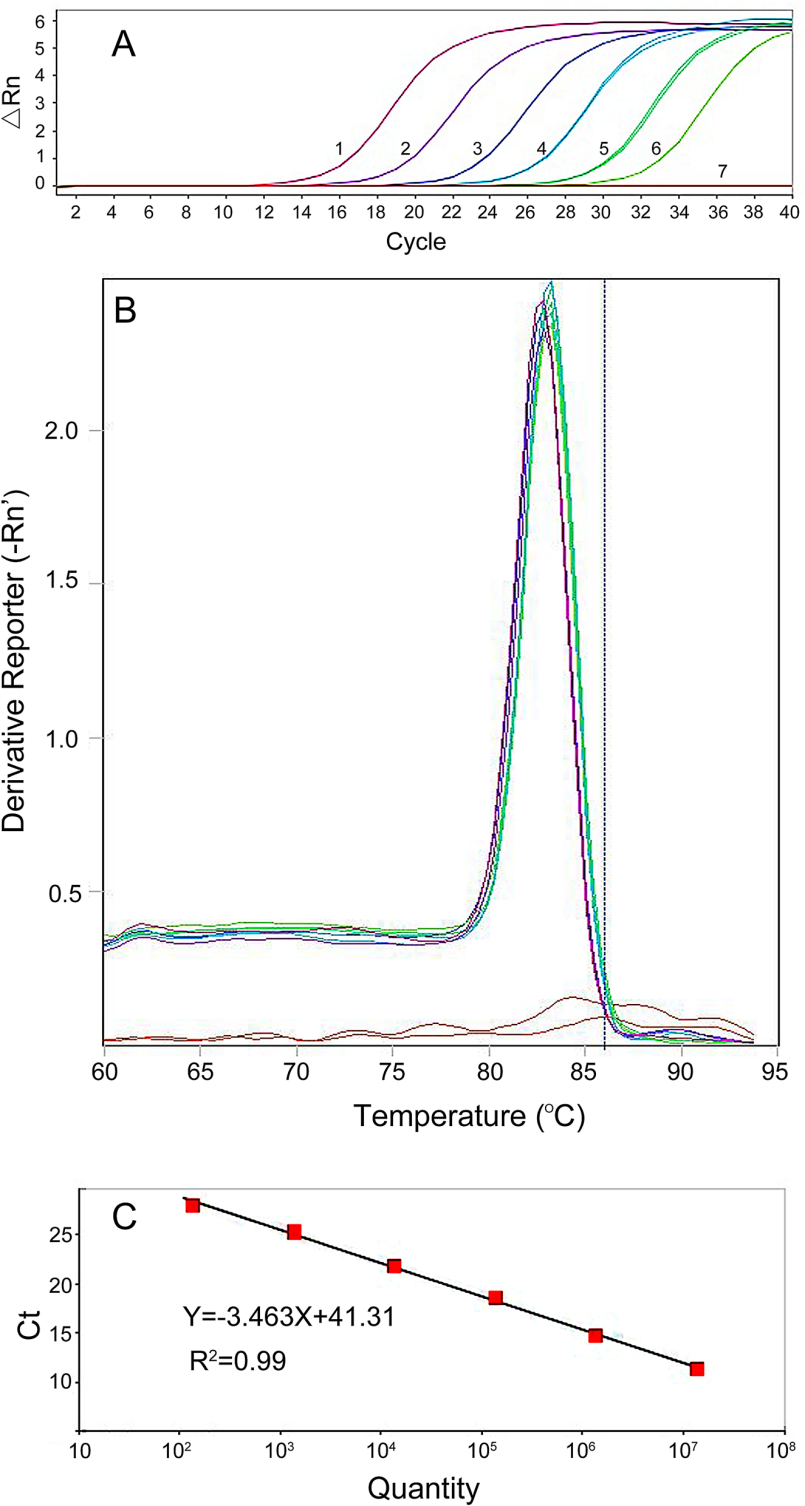
Establishment of a standard curve by SYBR Green I Real Time-PCR. (**A**) Real-time amplified curves. Lanes 1–6, tenfold dilutions of recombinant plasmid DNA (10 ng–100 fg); Lane 7 negative control ddH_2_O. (**B**) Melting curve of SYBR Green I. (**C**) Standard curve.

To improve the detection limit of the primers, we used real-time PCR with SYBR Green I in this study. Tenfold serial dilutions of plasmid DNA (CN = 8.29 × 10^9^–8.29 × 10^4^, 10 ng–100 fg) were used as a template (Fig. 5a). In addition, the standard curve was generated with a linear range covering 6 log units. The correlation coefficient of the standard curve reached 0.99, and the amplification efficiency was 107.3% (Fig. 5c). To demonstrate that the amplification was specific for the SCAR marker, we performed a melting curve analysis immediately after the real-time PCR analysis. Melting curve analysis showed that the SCAR marker only had one predominant peak (Fig. 5b). These results suggested that the SYBR Green I real-time PCR detection method for *T. laevis* was successfully established.

### Digital droplet PCR (ddPCR) detection

**Figure 6:**
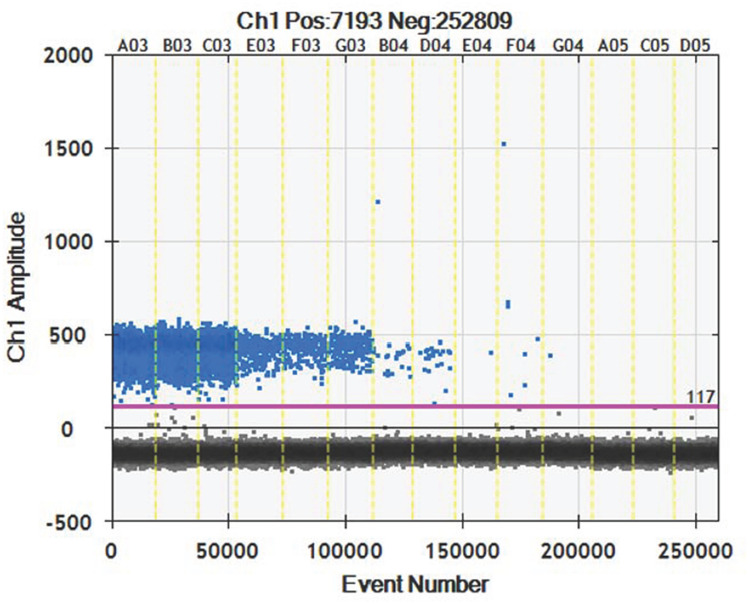
Distribution diagram of droplets of *T. laevis* and *T. controversa* isolates by droplet digital PCR. A03–C03, DNA template of *T. laevis* (30 ng/µl); E03–G03, DNA template of *T. laevis* (3 ng/µl); B04–E04, DNA template of *T. laevis* (0.3 ng/µl); F04–A05, DNA template of *T. laevis* (30 fg/µl), C05–D05, ddH_2_O control; blue dots are positive droplets, and black dots are negative controls.

**Figure 7:**
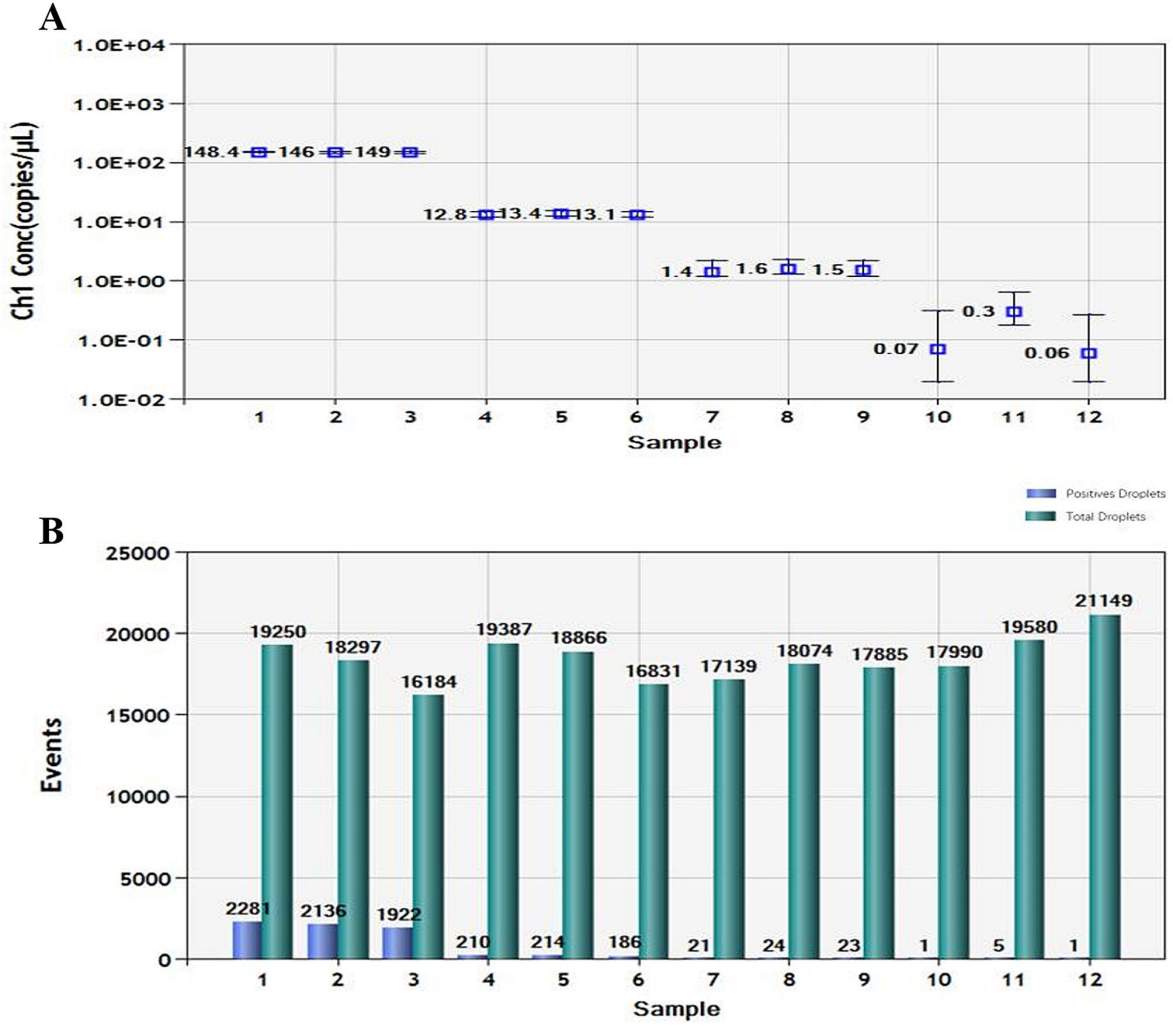
Statistical analysis by ddPCR (**A**) Positive copy number analysis for detection of *T. laevis* by copy number; 1–3, DNA template of *T. laevis* (30 ng/µl); 4–6, DNA template of *T. laevis* (3 ng/µl); 7–9, DNA template of *T. laevis* (0.3 ng/µl); 10–12, DNA template of *T. laevis* (30 fg/µl). (**B**) Number analysis of *T. laevis* isolates; 1–3, DNA template of *T. laevis* (30 ng/µl); 4–6, DNA template of *T. laevis* (3 ng/µl); 7–9, DNA template of *T. laevis* (0.3 ng/µl); 10–12, DNA template of *T. laevis* (30 fg/µl). Gray pillars are positive droplets, and blue pillars are total droplets (positive + negative).

For ddPCR, 10,000 droplets were used, which is a precise and reliable number. More blue droplet points indicate the presence of an increased number of positive droplets in a sample and thus a greater copy number in the ddPCR product and a higher concentration of *T. laevis* in the DNA sample. A zero-positive droplet means there was no detection of *T. laevis*. The results showed that a concentrated droplet fluorescence intensity was noted in most samples with a greater number of blue droplets. Additionally, there were no blue droplets in the *T. controversa* samples (Fig. 6). Furthermore, the results showed that the lowest concentration of 1.5 copies/µl (30 fg/µl) was detected by ddPCR in the *T. laevis* DNA, and statistical analysis of the positive droplet quantities demonstrated that ddPCR was effective and successful for detection of *T. laevis* DNA. The analysis of the total number of droplets is shown in Fig. 7. Based on the above results, ddPCR is more sensitive and can detect the lowest concentration of DNA compared to standard PCR and real-time PCR.

## Discussion

Internal transcribed spacer (ITS), specific DNA sequences, and DNA molecular marker technology (AFLP, RAPD, ISSR) have been widely used to identify *T. laevis*-related species, including *T. caries*, *T. controversa*, and *T. horrida*^5,12,16,34,35^. All these methods failed to differentiate *T. laevis* from *T. caries*. In this study, a species-specific SCAR marker of *T. laevis* was developed with the ISSR technique. We tested the specificity with its related genera and species, such as *T. laevis, T. controversa, T. caries, U. tritici, U. hordei, U. maydis, P. striiformis* f. sp. *tritici, P. graminis* f. sp*. tritici, P. triticina, R. cerealis, F. graminearum*, *B. graminis* f. sp. *tritici* and *B. sorokiniana.* The high specificity of the SCAR marker suggested that it could be used to accurately distinguish *T. laevis*. Although Zhang et al.^19^ developed a SCAR marker (286 bp) for the detection of *T. laevis* by AFLP, the sensitivity of the SCAR marker was not tested against *T. laevis*. The SCAR marker developed in this study from ISSR was 763 bp and could detect 5 ng/µl in a 25 µl PCR mixture. The 763 bp product is larger than the 286 bp product. Thus, for this procedure, it will be easier to run the gels after PCR and will save time.

Moreover, to further improve the sensitivity, we employed real-time PCR with SYBR Green I. Our real-time PCR results showed higher sensitivity than that of the SCAR marker with standard PCR, which was similar to other studies^33^. Recent advances in molecular detection and quantification have showed that standard PCR, SCAR markers and real-time PCR are highly efficient for pathogen detection^16^^.^ Yao et al. developed a SCAR marker for *T. laevis* with a detection limit of 0.4 ng/μl of DNA from *T. laevis,* and a SYBR Green I real-time PCR method was also successfully developed based on the SCAR marker with a detection limit of 10 fg/μl *T. laevis* DNA^20^^.^ In this study, the sensitivity of real-time PCR was 100 fg/µl, which was much more sensitive than that of traditional PCR detection methods (5 ng/µl).

DdPCR can achieve accurate quantification of plant pathogens without standards and is the latest and most advanced technology that is shows promise for calibration of reference materials worldwide. DdPCR can be used for the identification and quantification of pathogens, such as *T. controversa*^33^, which demonstrated a detection sensitivity of 2.1 copies/µl, and the results in this study showed that ddPCR could detect 30 fg/µl (1.5 copies/µl) of *T. laevis* DNA. DdPCR has already been successfully used for the detection of other pathogens^31,36–38^ and plant pathogens, such as *Phytoplasma*^39^, *Erwinia amylovora* and *Ralstonia solanacearum*^40^. Therefore, ddPCR has good potential for practical use in plant pathogen detection, especially for detection of quarantine organisms with small samples, even though running cost remains slightly above that of real-time PCR.

In summary, we developed ddPCR detection methods based on SCAR marker derived from ISSR, and real-time PCR with SYBR Green I for rapid and accurate detection of *T. laevis*. The obtained results from our study support the use of the ddPCR detection method in place of the SCAR marker and real-time PCR for sensitivity and accuracy of *T. laevis* detection. This study is the first to detect *T. laevis* teliospores with enhanced sensitivity of ddPCR techniques.

## Materials and methods

### Fungal isolates and DNA extraction

Isolates of *T. laevis, T. controversa, T. caries, U. tritici, U. hordei, U. maydis, P. striiformis* f. sp. *tritici, P. graminis* f. sp*. tritici, P. triticina, R. cerealis, F. graminearum*, *B. graminis* f. sp. *tritici* and *B. sorokiniana* were used in this study. The origin and number of the strains are listed in Table S1. DNA was extracted from urediniospores for *P. striiformis* f. sp. *tritici*, *P. triticina*, and *P. graminis* f. sp. *tritici*, from conidia for *B. graminis*, from teliospores for *T. laevis*, *T. controversa*, *T. caries*, *U. tritici*, *U. hordei*, *U. maydis*, and from vegetative hyphae for *R. cerealis*, *F. graminearum* and *B. sorokiniana* which were cultured on potato dextrose agar (PDA). Genomic DNA of all isolates (20 mg urediniospores, conidia or teliospores, and 3 plates of vegetative hyphae on PDA for each isolate) were extracted using a protocol reported by Liu^17^ with slight modification (material were crushed with FastPrep-24 (MP, USA). DNA quantitation and purity were assessed by Nanodrop 2000 (Thermo Scientific, Waltham, USA) with absorbance ratios (OD_260_/OD_280_ between 1.8–2.0). DNA integrity was checked by agarose gel electrophoresis with λDNA/*Hin*dIII marker. Then, the DNA were stored at − 20 °C for further use.

### ISSR-PCR amplification

For identification of the different fragments, the extracted genomic DNA of *T. laevis* and other common wheat pathogens were amplified using one hundred ISSR primers, which were designed by the University of British Columbia (https://www.michaelsmith.ubc.ca/services/NAPS/Primer_Sets). All primers were synthesized by Sangon Biological Engineering Technology and Services Co., Ltd. (Shanghai, China). The total reaction system volume was 25 µl, including 12.5 µl of 2 × PCR SuperMix (TransGen Biotech, Beijing, China), 2 µl of ISSR primer (10 μM), 1 µl of template DNA (100 ng/µl) and 9.5 µl of ddH_2_O (Tiangen Biotech, Beijing, China). Amplification was carried out in a programmable optics module thermocycler (Bio-Rad, USA). The PCR cycling conditions were as follows: initial denaturation at 95 °C for 3 min; 30 cycles of denaturation at 95 °C for 1 min, annealing at 40–75 °C (depending on the primer) for 30 s, and extension at 72 °C for 2 min; followed by a final extension step at 72 °C for 5 min. The amplified products were tested by running on 1.5% agarose gel electrophoresis containing ethidium bromide, and the expected bands were visualized using the gel documentation system (WSE-5200 Printgraph 2 M, ATTO, Korea) as described previously^16^^.^

### Cloning the species-specific DNA fragment and SCAR marker development

The specific band (1385 bp) of the *T. laevis* DNA generated by the primer ISSR857 (5´-ACACACACACACACA-3´) was excised from the gel, purified with the EasyPure Quick Gel Extraction Kit (TransGen Biotech, China), and ligated into the pMD18-T vector using a cloning kit (TaKaRa, Japan). The cloned fragment was sequenced, and a pair of SCAR marker primers (L57F:5′-CGAGTGCTCTTGGTGGGAAT-3′/L57R:5′-GCGAGGCGTTTTCACAGTTT-3′) was designed and synthesized by Sangon Biological Engineering Technology, Ltd. (Beijing, China).

### Specificity of the SCAR marker

The specificity of the SCAR marker was determined with genomic DNA based on three factors^20^, excluding the genomic DNA of *T. laevis*. First, DNA from the fungal species that shared similarity with *T. laevis,* including *T. controversa* and *T. caries*, was used. Second, we selected DNA from pathogens that cause disease in the leaves and tassels of wheat, such as *P. triticina, P. striiformis* f. sp. *tritici, P. graminis* f. sp. *tritici, R. cerealis, F. graminearum, B. graminis* f. sp. *tritici* and *B. sorokiniana*. Finally, we selected DNA from pathogens that caused smut diseases in cereal crops, including *U. tritici, U. hordei* and *U. maydis*. SCAR amplification was performed in a total volume of 25 µl, including 12.5 µl of 2 × PCR SuperMix (TransGen Biotech, Beijing, China), 1 µl of SCAR L57F primer (10 µM), 1 µl of SCAR L57R primer (10 µM), 1 µl of DNA template (100 ng/µl) and 9.5 µl of ddH_2_O (Tiangen Biotech, Beijing, China). PCR amplification was performed as follows: initial denaturation at 94 °C for 5 min; 30 cycles of denaturation at 94 °C for 30 s, annealing at 57 °C for 30 s, and extension at 72 °C for 30 s; followed by a final extension step at 72 °C for 10 min. The amplified products were separated by 1.5% agarose gel electrophoresis with GelStain (1000 X) (TransGen, Biotech, China) at 150 V for 30 min in 0.5 × TBE buffer and then visualized using the gel documentation system (WSE-5200 Printgraph 2 M, ATTO, Korea).

### Sensitivity of the SCAR marker

The sensitivity of the SCAR marker was tested with purified genomic DNA of *T. laevis*, which was serially diluted at the following concentrations: 50 ng, 25 ng, 10 ng, 5 ng, 1 ng, 100 pg 10 pg, 1 pg and 0.1 pg in 25 μl of PCR mixture. The PCR mixture, amplification procedure and agarose gel electrophoresis conditions were the same as those mentioned above.

### Real-time PCR detection method

The primer pair (5′-ATCATTCTTGCGGCGAACA-3′ and 5′-GATCACAGCATCCACGAGACA-3′) was derived from SCAR and synthesized by Sangon Biological Engineering Technology, Ltd. (Beijing, China). Real-time PCR was performed in a total reaction volume of 20 µl containing 10 µl of Green qPCR SuperMix (+ Dye II) (TransGen Biotech, Beijing , China), 0.4 µl of forward primer (10 µM), 0.4 µl of reverse primer (10 µM), 2 µl of plasmid DNA sample and 7.6 µl of ddH_2_O (Tiangen Biotech, Beijing, China). After the reaction system was well mixed and centrifuged, aliquots were loaded onto a 96-well PCR plate (Eppendorf, Germany). Three biological and technical replicates of real-time PCR were designed for each sample, and 2 µl of nuclease-free water (Tiangen Biotech, Beijing, China) was used as a control on each plate. Compared to the protocols from Yao et al*.*^15^, the ABI 7500 real-time PCR system (Applied Biosystems, Carlsbad, CA, USA) was used with the same reaction program settings except annealing at 57 °C for 40 s and the same settings for generation of melt curves and collection of the fluorescent signal.

### Droplet digital PCR (ddPCR) detection method

The DNA used for the method was assessed as described by Liu et al*.*^33^. Based on the SCAR marker and real-time PCR for *T. laevis*, we developed the primers for droplet digital PCR (ddPCR). The primers (forward: 5′-GTATGGCCGACACGAATCTAG-3′, reverse: 5′-TCGGAGCAAAAGATCATGGG-3′) and probe (FAM 5′-TGAGCAAGAGTGAAGCCTCAAAAGGG-3′ TAMRA) were synthesized by Sangon Biological Engineering Technology and Services Co., Ltd. (Shanghai, China). The ddPCR reaction mix was composed of 20 μl of ddPCR Mix for Probes, 1.8 μl of forward primer (10 μM), 1.8 μl of reverse primer (10 μM), 0.5 μl of probe (10 μM), 2.0 μl of DNA template (10 ng/μl), and 3.9 μl of ddH_2_O. We prepared the droplets using droplet-generating cards (186-4007, Bio-Rad, Hercules, USA) and a droplet generator (QX200, Bio-Rad, USA). Forty microliters of PCR master mix and 70 μl of droplet-generating oil (186-3005, Bio-Rad, Hercules, USA) were added to a droplet-generating card to generate droplets in the droplet generator. Three biological and technical replicates were designed for each sample. We transferred the droplet emulsion to a new 96-well PCR plate (Eppendorf) and amplified it in a C1000 Touch Thermal Cycler (Bio-Rad). ddPCR was carried out with the following program settings: initial denaturing at 98 °C for 10 min followed by 40 cycles of denaturing at 94 °C for 30 s, annealing at 55 °C for 1 min, and extension at 98 °C for 10 min. Then, the plates were moved to a droplet reader (QX200, Bio-Rad, Hercules, CA, US), and data were obtained based on the analysis by Quanta Soft (Version, 1.7.4, Bio-Rad, provided with the ddPCR system) analysis^22^. The experiments were repeated three times. We also used the JavaScript program “dedinetherain” to set the threshold florescence amplitude, with the aim of providing a better estimate of the number of positive and negative droplets and increasing the reproducibility of the results as Liu et al.^33^.

## Supplementary information

Supplementary Information.
