# Supplementary material for: Development of droplet digital PCR for the detection of Tilletia laevis, which causes common bunt of wheat, based on the SCAR marker derived from ISSR and real-time PCR
Source: Sci Rep. 2020 Sep 30;10:16106. doi: 10.1038/s41598-020-72976-7 (PMC7528053; doi:10.1038/s41598-020-72976-7)
Supplement: Supplementary file 1 — Supplementary Information. [file 41598_2020_72976_MOESM1_ESM.docx]

 Title page for Supplementary Information

**Development of droplet digital PCR for the detection of *Tilletia laevis*, which causes common bunt of wheat, based on the SCAR marker derived from ISSR and real-time PCR**

Tongshuo Xu^1^, Zhaoqun Yao^1,2^, Jianjian Liu^1,3^, Han Zhang^1,2^, Ghulam Muhae Ud Din^1^, Sifeng Zhao^2^, Wanquan Chen^1^, Taiguo Liu^1^, Li Gao^1*^

^1^State Key Laboratory for Biology of Plant Disease and Insect Pests, Institute of Plant Protection, 100193, Beijing, China

^2^Key Laboratory at Universities of Xinjiang Uygur Autonomous Region for Oasis Agricultural Pest Management and Plant Protection Resource Utilization, Shihezi University, 832003, Xinjiang, China

^3^School of Agriculture, Yangtze University, 434023, Hubei, China.

*Correspondence: Li Gao ([xiaogaosx@hotmail.com](mailto:xiaogaosx@hotmail.com))

**Table S1 The information of the isolates used in this study**

| **Isolates** | **Number** | **Origin** | **Isolated and identified information** |
| --- | --- | --- | --- |
| *Tilletia laevis* |  | Henan, China | Prof. Li Gao |
| *Tilletia controversa* |  | USDA-ARS | Gift from Mr Blair Goates |
| *Tilletia caries* |  | Henan, China | Prof. Li Gao |
| *Ustilago tritici* |  | Gansu, China | Prof. Li Gao |
| *Ustilago hordei* |  | Qinghai, China | Prof. Li Gao |
| *Ustilago maydis* |  | Beijing, China | Prof. Wang Xiaoming (Institute of Crop Science, CAAS) |
| *Puccinia striiformis* f. sp. *tritici* |  | Gansu, China | Prof. Wanquan Chen |
| *Puccinia graminis* f.sp*. tritici,* |  | Shenyang, China | Prof. Wanquan Chen |
| *Puccinia triticina* |  | Hebei, China | Prof. Wanquan Chen |
| *Rhizoctonia cerealis* |  | Henan, China | Prof. Li Gao |
| *Fusarium graminearum* |  | Jiangsu, China | Prof. Li Gao |
| *Blumeria graminis* f. sp. *tritici* |  | Henan, China | Prof. Li Gao |
| *Bipolaris sorokiniana* |  | Henan, China | Prof. Li Gao |

**Table S2. Primers used in the study**

| **Name** | **Primer sequences** |
| --- | --- |
| ISSR857 | 5´-ACACACACACACACA-3´ |
| SCAR marker | L57F:5'-CGAGTGCTCTTGGTGGGAAT-3')/L57R:5'-GCGAGGCGTTTTCACAGTTT-3') |
| Real-time PCR | 5'-ATCATTCTTGCGGCGAACA-3'/5'-GATCACAGCATCCACGAGACA-3' |
| DdPCR | 5'-GTATGGCCGACACGAATCTAG-3'/5'-TCGGAGCAAAAGATCATGGG-3'  Probe: FAM 5'-TGAGCAAGAGTGAAGCCTCAAAAGGG-3' TAMRA |
